# Documenting Penicillin Allergy: The Impact of Inconsistency

**DOI:** 10.1371/journal.pone.0150514

**Published:** 2016-03-16

**Authors:** Nirav S. Shah, Jessica P. Ridgway, Natasha Pettit, John Fahrenbach, Ari Robicsek

**Affiliations:** 1 Department of Medicine, University of Chicago, Chicago, Illinois, United States of America; 2 Department of Clinical Analytics, Northshore University HealthSystem, Evanston, Illinois, United States of America; 3 Department of Medicine, Northshore University HealthSystem, Evanston, Illinois, United States of America; Chang-Gung University, TAIWAN

## Abstract

**Background:**

Allergy documentation is frequently inconsistent and incomplete. The impact of this variability on subsequent treatment is not well described.

**Objective:**

To determine how allergy documentation affects subsequent antibiotic choice.

**Design:**

Retrospective, cohort study.

**Participants:**

232,616 adult patients seen by 199 primary care providers (PCPs) between January 1, 2009 and January 1, 2014 at an academic medical system.

**Main Measures:**

Inter-physician variation in beta-lactam allergy documentation; antibiotic treatment following beta-lactam allergy documentation.

**Key Results:**

15.6% of patients had a reported beta-lactam allergy. Of those patients, 39.8% had a specific allergen identified and 22.7% had allergic reaction characteristics documented. Variation between PCPs was greater than would be expected by chance (all p<0.001) in the percentage of their patients with a documented beta-lactam allergy (7.9% to 24.8%), identification of a specific allergen (e.g. amoxicillin as opposed to “penicillins”) (24.0% to 58.2%) and documentation of the reaction characteristics (5.4% to 51.9%). After beta-lactam allergy documentation, patients were less likely to receive penicillins (Relative Risk [RR] 0.16 [95% Confidence Interval: 0.15–0.17]) and cephalosporins (RR 0.28 [95% CI 0.27–0.30]) and more likely to receive fluoroquinolones (RR 1.5 [95% CI 1.5–1.6]), clindamycin (RR 3.8 [95% CI 3.6–4.0]) and vancomycin (RR 5.0 [95% CI 4.3–5.8]). Among patients with beta-lactam allergy, rechallenge was more likely when a specific allergen was identified (RR 1.6 [95% CI 1.5–1.8]) and when reaction characteristics were documented (RR 2.0 [95% CI 1.8–2.2]).

**Conclusions:**

Provider documentation of beta-lactam allergy is highly variable, and details of the allergy are infrequently documented. Classification of a patient as beta-lactam allergic and incomplete documentation regarding the details of the allergy lead to beta-lactam avoidance and use of other antimicrobial agents, behaviors that may adversely impact care quality and cost.

## Introduction

Documenting a history of medication allergy is an important task for any clinician. Ambiguities in a patient’s history can complicate this process, and likely contribute to patients being labeled as allergic to medications that they can actually tolerate safely. An example of this is allergy to beta-lactam antibiotics—the most common form of medication allergy in medical records of U.S. patients, with rates of documented penicillin allergy between 8–12% [1–4]. Despite this high prevalence, most patients with a history of a penicillin allergy are not actually allergic via penicillin skin testing and tolerate a penicillin challenge [4–12]. The frequent alternative to performing skin testing is to prescribe a non-penicillin antibiotic [13–14]. Broad spectrum fluoroquinolones, clindamycin, vancomycin and third-generation cephalosporins are commonly substituted for first-line narrow spectrum penicillins [15–18]. Older generation cephalosporins tend to cross react with penicillins more than structurally distinct third generation cephalosporins [7]. Avoidance of penicillins and first generation cephalosporins in beta-lactam allergic patients has been associated with greater lengths of stay, increased costs of care and increased rates of *Clostridium difficile*, vancomycin-resistant *Enterococcus* and methicillin-resistant *Staphylococcus aureus* (MRSA) [15–16,19].

History is an essential component to the beta-lactam allergy evaluation and determines the likelihood of a true allergy [20]. In the absence of a detailed history and a critical evaluation of the reaction, many patients may be mislabeled as allergic to beta-lactam antibiotics [21–24]. Despite the importance of the allergy history, incomplete documentation is widespread with studies showing incomplete documentation of allergic reactions ranging from 66–84% [15,17,25–29]. Inter-provider variation in allergy documentation has also been noted. In a study evaluating 834 patient electronic medical records receiving care from 167 ambulatory physicians, Soto et al. found that female internists were less likely than their male counterparts to document a drug allergy despite the presence of an electronic medical record designed to facilitate documentation [30]. The extent of inter-provider variation in allergy documentation is not well understood and the consequences of missing or non-specific allergy documentation on the subsequent use of antibiotics have not been well studied.

Using a comprehensive electronic health record (EHR) database we performed a retrospective review of all patients seen in a large healthcare system to evaluate for inter-provider variability in the labeling of patients with beta-lactam allergies and in documenting details of their reactions. We also examined how different forms of documentation affected subsequent antimicrobial prescriptions.

## Materials and Methods

NorthShore University HealthSystem (NorthShore) is a healthcare network in metropolitan Chicago with 4 hospitals and more than 50 outpatient clinics that employs over 850 physicians who use a single EHR platform (Epic, Epic Systems, Verona WI). Data from the EHR are housed in an enterprise data warehouse (EDW), which served as the source of data for this work. This study was approved by NorthShore’s Institutional Review Board. The requirement for informed consent was waived.

We collected data from the EDW on every patient greater than 17 years of age who was seen at an outpatient clinic by one of 199 NorthShore primary care providers (PCPs) specializing in internal medicine or family medicine from January 1, 2009 to January 1, 2014. For each patient, we collected demographics, all data entered in the EHR’s ‘Allergy Activity’, ICD9 diagnosis data and all medication prescription and administration data (inpatient and outpatient). We determined whether the patient was labeled as having beta-lactam allergy, and whether three features of beta-lactam documentation were present: 1) a specific agent was identified (e.g. amoxicillin, ceftriaxone as opposed to the more generic “penicillins” or “cephalosporins”); 2) the characteristics of the reaction were documented and 3) characteristics of a high risk reaction were noted. High risk characteristics included IgE-mediated reactions (e.g. anaphylaxis, angioedema, urticaria), documentation consistent with symptoms and signs of an IgE-mediated reaction (e.g. facial swelling, mouth swelling, shortness of breath, dyspnea), Stevens Johnson’s syndrome, exfoliative dermatitis, acute interstitial nephritis, hemolytic anemia, agranulocytosis, serum sickness and hepatitis. Each documented reaction was individually reviewed by an investigator to determine if these high risk features were present. Patients were considered to be in a physician’s panel if they had that provider designated as their PCP in the EHR and were seen by the provider at least once. Patients are linked to only one provider and patients without a NorthShore PCP were excluded. Of note, patients also acquired allergy documentation from other providers in the health system. We calculated the percentage of all allergies that were documented by PCPs.

For each physician, we measured the percentage of patients in their panel who had a beta-lactam allergy noted, the percentage of these cases where a specific allergen was identified, the characteristics of the reaction were documented and high-risk characteristics were present. We used large volume providers (panels greater than 1,000 unique patients or greater than 100 patients with a beta-lactam allergy) to reduce random chance effects of documentation that may have occurred with low volume providers. In using this cutoff, we did not assume that low volume providers document differently than high volume providers. For statistical analysis, binomial distribution was assumed to generate confidence intervals. To determine whether inter-provider variability was greater than that expected by chance we simulated expected frequency distributions for variation in beta-lactam allergy documentation and our three features of beta-lactam allergy documentation among providers. For the simulation, we assumed documentation events for each patient were independent of the events for other patients and that the event documentation rate for each physician was independent of other physicians. To run each simulation, the patient panel size of each physician was held constant at its true observed size (e.g. a physician with 1000 patients in their panel was assigned 1000 patients in each simulation) and their patients were randomly assigned as either having or not having the event based on the global event rate of all the patients in the study. The percentage of events in each physician’s patient panel was then calculated and the simulation was repeated 100 times. After all the simulations were completed, the distribution of the percentage of events in each physician’s patient panel was calculated. This expected distribution was compared to the observed distribution of events in each physician’s patient panel. Levene’s test was used to compare the equality of variance between the observed and expected distributions.

For all patients with beta-lactam allergy, we determined the first antibiotic prescribed as either an inpatient or outpatient following the date of beta-lactam allergy documentation. For patients without a beta-lactam allergy we determined the first antibiotic prescribed during the study period. We compared first antibiotic prescribed among patients with and without a beta-lactam allergy. We further compared first antibiotic prescribed among patients with and without a specific allergen identified, an allergic reaction documented and a high risk reaction present. A comparison of first antibiotic prescribed among patients with and without a beta-lactam allergy divided by age classification is included in S1 Table. We also evaluated first antibiotic prescription among patients with and without beta-lactam allergies who were diagnosed with celllulitis (as noted by ICD9 code) during outpatient or inpatient encounters (S2 Table). We reported the top six classes of antibiotics that were most likely to be affected by beta lactam allergy documentation.

Student t tests were used to compare means and proportions and Chi square statistics were used to compare observed and expected frequency distributions. Relative risks were calculated. P values < 0.05 were considered statistically significant. The simulated probability distribution curves for each category were generated in R and results were analyzed with Stata (Version 12, StataCorp, College Station, Texas) and R: A Language and Environment for Statistical Computing (R Foundation for Statistical Computing, 2014, Vienna, Austria).

## Results

We identified 232,616 unique patients cared for by 198 PCPs at NorthShore during the study period. Of these patients, 36,193 patients (15.6%) had a documented beta-lactam allergy of which 14.7% and 1.5% were attributable to penicillins and cephalosporins, respectively (0.6% of patients had both). Of the beta-lactam allergic patients, 14,415 (39.8%) had a specific beta-lactam allergen identified and 8,226 (22.7%) had characteristics of the beta-lactam allergy documented. Of the 8,226 patients with a beta-lactam reaction documented 1,361 (16.5%) had high-risk characteristics (Table 1).

**Table 1:** Baseline characteristics.

Documentation (N)	N (%)	Mean Age—yr	Female sex—No. (%)	Male sex—No. (%)	Race (Caucasian) No. (%)
**ALL PATIENTS (232,616)**					
Beta-lactam allergy	36,193 (15.6)	51.0*	24,020 (66.4)*	12,173 (33.6)*	25,588 (70.7)*
No beta-lactam allergy	196,423 (84.4)	47.2*	104,491 (53.2)*	91,932 (46.8)*	118,618 (60.4)*
**WAS A SPECIFIC BETA- LACTAM ALLERGEN IDENTIFIED? (36,193)**					
Specific beta-lactam	14,415 (39.8)	48.7*	9,761 (67.7)*	4,654 (32.3)*	10,381 (72.0)*
No specific beta-lactam	21,778 (60.2)	52.6*	14,259 (65.5)*	7,519 (34.5)*	15,207 (69.8)*
**WERE CHARACTERISTICS OF BETA-LACTAM REACTION DOCUMENTED? (36,193)**					
Documented	8,226 (22.7)	52.3*	5,442 (66.2)	2,784 (33.8)	5,993 (72.9)*
Not documented	27,967 (77.3)	50.7*	18,578 (66.4)	9,389 (33.6)	19,595 (70.1)*
**WAS BETA-LACTAM REACTION HIGH-RISK? (8,226)**					
High risk reaction	1,361 (16.5)	51.6	952 (70.0)^ǂ^	409 (30.0)^ǂ^	973 (71.5)
Not a high risk reaction	6,865 (83.5)	52.4	4,490 (65.4)^ǂ^	2,375 (34.6)^ǂ^	5,020 (73.1)

*p < 0.001;

^ǂ^ p < 0.01

In univariable analysis, patients who had a beta-lactam allergy were more likely to be older, female and white (p < 0.001). Among those with a beta-lactam allergy, younger age, female sex and white race were associated with identification of a specific beta-lactam allergen (p < 0.001); older age and white race were associated with having characteristics of the beta-lactam reaction documented (p < 0.001); female sex was associated with high risk reactions (p = 0.001) (Table 1).

**Fig 1:**
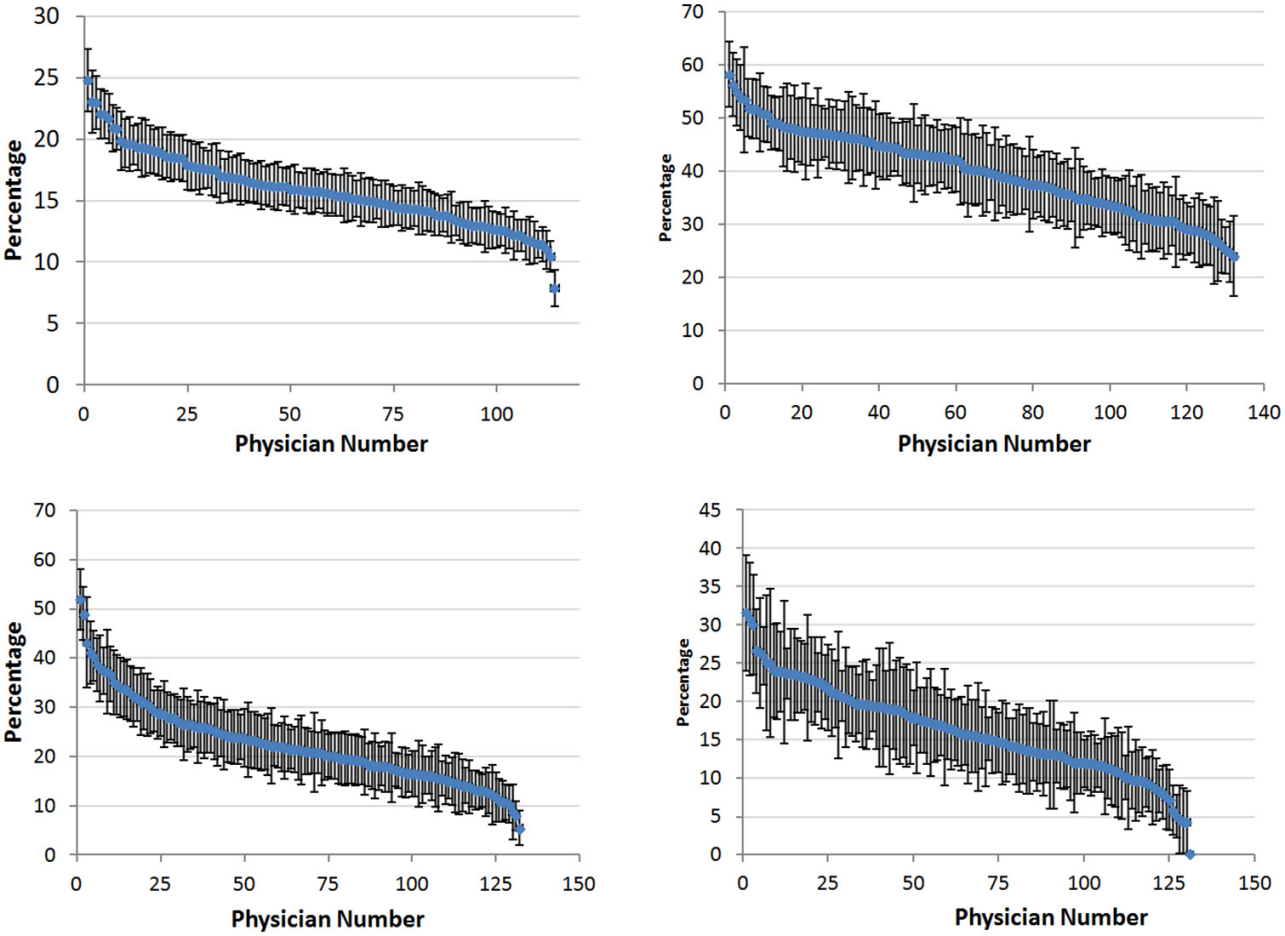
a-d. Percentage of patients with a beta-lactam allergy (Fig 1a), with a specific beta-lactam allergen identified (Fig 1b), with characteristics of a beta-lactam reaction documented (Fig 1c) and with a high risk beta-lactam reaction documented (Fig 1d) by high volume primary care provider panels. Binomial distribution was assumed to generate confidence intervals.

There was high variability in beta-lactam allergy documentation across PCP panels. Among the 114 highest volume physicians (with panels of >1,000 unique patients) these rates varied from 7.9% to 24.8% (Fig 1a). For the 131 providers with patient panels with greater than 100 beta-lactam allergic patients, high variability was also seen in the rates at which a specific beta-lactam allergen was identified (24.0% to 58.2%), characteristics of the reaction were documented (5.4% to 51.9%) and high risk reaction characteristics were noted (0% to 31.6%) (Fig 1b–1d). Notably, of the 36,193 patients with a beta-lactam allergy only 6,218 (17.2%) of these patients had the allergy documented by their PCP.

**Fig 2:**
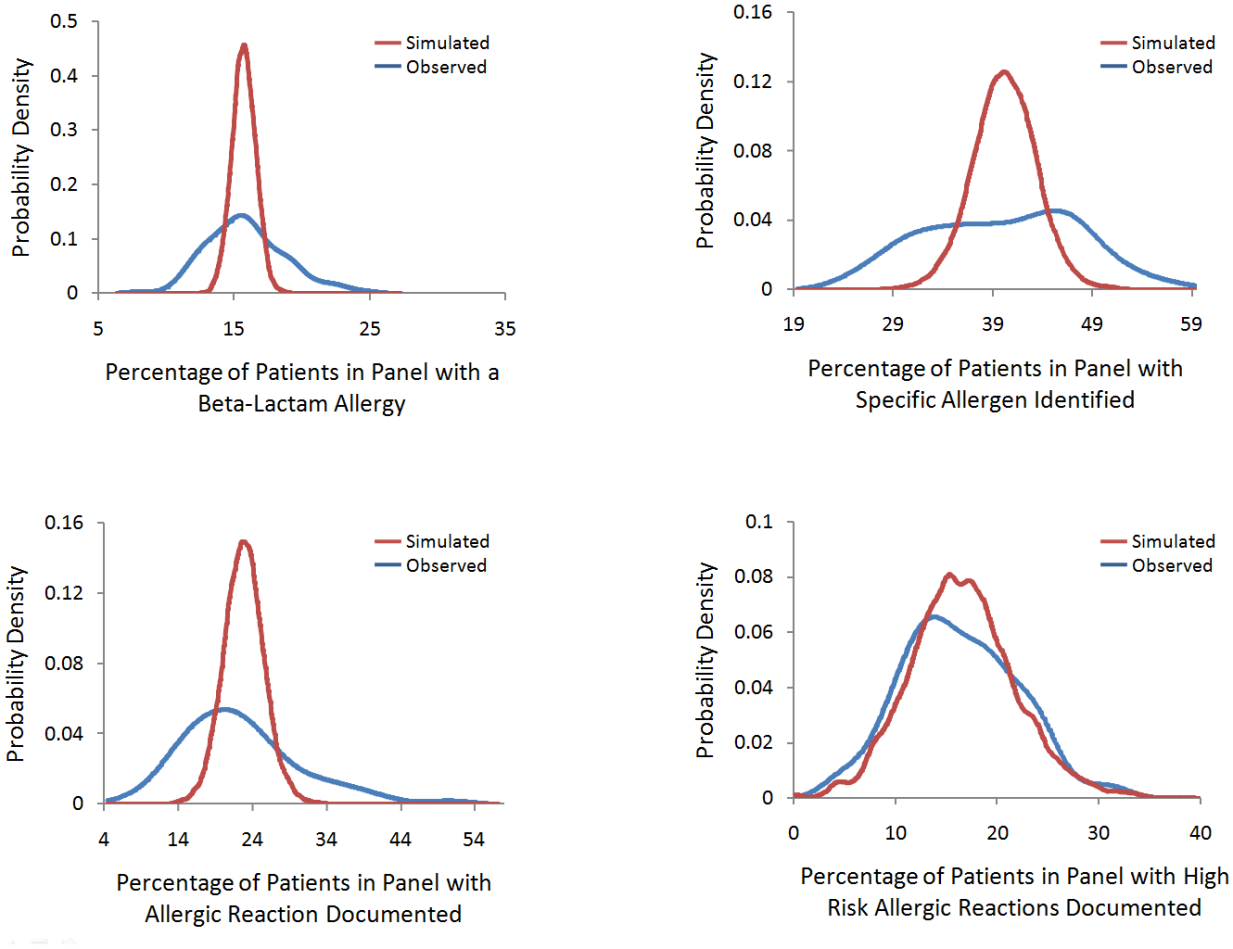
a-d. Observed vs. simulated expected frequency distribution of the percentage of patients with a beta-lactam allergy (Fig 2a), a specific beta-lactam allergen identified (Fig 2b), with characteristics of a beta-lactam reaction documented (Fig 2c) and with a high-risk beta-lactam allergic reaction documented (Fig 2d) by physician cohort. In the simulation, expected distribution was determined by holding patient panel size constant and randomly assigning patients as either having or not having the event based on the global event rate of all the patients in the study. The simulation was repeated 100 times and the distribution of the percentage of events in each physician’s patient panel was calculated. Levene’s test was used to compare the equality of variance between the observed and expected distributions.

To determine whether the variability in documentation would be expected by chance (i.e. based only on chance differences in the panels cared for by different physicians) we compared simulated to observed frequency distributions for beta-lactam allergy documentation by physician (Fig 2a–2d). The observed distributions differed significantly from those expected for beta-lactam allergy documentation, specific allergen identification and documentation of the reaction’s characteristics (p<0.001). The observed distribution was not significantly different from the expected distribution for high risk characteristics documented (p = 0.06).

To evaluate how beta-lactam documentation affected subsequent antimicrobial therapy, we followed all patients from the time of allergy documentation until the end of the study period (median 2,447 days, range 653 to 3,958 days) and identified the first antibiotic (inpatient or outpatient) which they subsequently received. Patients with a documented beta-lactam allergy were less likely to receive penicillins (3.5% vs 21.9%; RR 0.16 [95% CI: 0.15–0.17]) and cephalosporins (4.7% vs 16.6%; RR 0.28 [95% CI 0.27–0.30]) but were more likely to receive fluoroquinolones (24.7% vs 16.4%; RR 1.5 [95% CI 1.5–1.6]), clindamycin (11.0% vs 2.9%; RR 3.8 [95% CI 3.6–4.0]), vancomycin (1.5% vs 0.3%; RR 5.0 [95% CI 4.3–5.8]) and macrolides (43.5% vs 33.1%; RR 1.3 [95% CI 1.29–1.34]). Patients with a specific allergen identified were more likely to receive penicillins (5.3% vs 2.2%; RR 2.4 [95% CI 2.1–2.8]) and cephalosporins (5.3% and 4.3%; RR 1.2 [95% CI 1.1–1.4]) and less likely to receive fluoroquinolones (23.5% vs 25.8%; RR 0.91 [95% CI 0.87–0.96]) and clindamycin (10.3% vs 11.5%; RR 0.90 [95% CI 0.83–0.97]) than those with beta-lactam allergy but without a specific allergen identified. Patients with characteristics of their reaction documented were more likely to receive penicillins (6.1% vs 2.8%; RR 2.2 [95% CI 1.9–2.5]) and cephalosporins (7.3% vs 4.0%; RR 1.8 [95% CI 1.6–2.1]). Characteristics of high risk reactions were associated with fewer penicillins (3.6% vs 6.6%; RR 0.54 [95% CI 0.35–0.82]) and cephalosporins (4.2% vs 7.8%; RR 0.54 [95% CI 0.37–0.79]) but more fluoroquinolones (28.0% vs 23.7%; RR 1.2 [95% CI 1.0–1.4]) (Table 2). Patients with beta-lactam allergy as compared to those without beta-lactam allergy were less likely to receive penicillins, cephalosporins and more likely to receive fluoroquinolones, clindamycin, vancomycin and macrolides for all age groups (S1 Table). When looking only at unique patients with a clinical encounter for cellulitis (by ICD9 code), patients with beta-lactam allergy as compared to those without beta-lactam allergy were less likely to receive penicillins and cephalosporins and more likely to receive fluoroquinolones, clindamycin, vancomycin and macrolides (S2 Table). Patients with cellulitis with a specific beta-lactam allergen identified were more likely to receive a penicillin than those patients without a specific beta-lactam allergen identified.

**Table 2:** Among patients who received an antibiotic, first antibiotic prescribed after antibiotic allergy documentation^1^^,^^2^.

Documentation (N)	Penicillins (%)	Cephalosporins (%)	Fluoroquinolones (%)	Clindamycin (%)	Vancomycin (%)	Macrolides (%)
**ALL PATIENTS (161,658)**						
Beta-lactam allergy (21,196)	3.5*	4.7*	24.7*	11.0*	1.5*	43.5*
No beta-lactam allergy (140,462)	21.9*	16.6*	16.4*	2.9*	0.3*	33.1*
**WAS A SPECIFIC BETA- LACTAM ALLERGEN IDENTIFIED? (21,196)**						
Specific beta-lactam (8,811)	5.3*	5.3*	23.5*	10.3ǂ	1.5	43.0
No specific beta-lactam (12,302)	2.2*	4.3*	25.8*	11.5ǂ	1.4	43.5
**WERE CHARACTERISTICS OF BETA-LACTAM REACTION DOCUMENTED? (21,196)**						
Documented (4,403)	6.1*	7.3*	24.2	10.4	1.2	40.3*
Not documented (16,793)	2.8*	4.0*	24.8	11.1	1.5	44.4*
**WAS BETA-LACTAM REACTION HIGH RISK? (4,403)**						
High risk reaction (646)	3.6^ǂ^	4.2^ǂ^	28.0^Ϯ^	12.0	1.1	41.8
Not a high risk reaction (3,660)	6.6^ǂ^	7.8^ǂ^	23.7^Ϯ^	10.2	1.2	39.9

*p < 0.001;

^ǂ^ p < 0.01;

^Ϯ^ p < 0.05

^1^ Rows do not add up to 100 because other antibiotic classes not displayed

^2^ Only patients who received an antibiotic after allergy documentation were included. For patients who did not have a beta-lactam allergy documented, the first antibiotic prescribed is the first antibiotic received during the study period

## Discussion

An accurate and complete allergy history is an essential tool in antimicrobial decision-making. The present investigation was designed to evaluate whether physicians differ in how they document this history, and how this variability may affect subsequent clinical decision-making. In this population-based study of 232,616 adults across a health network, beta-lactam allergy documentation was highly variable and often incomplete.

Overall, we found a beta-lactam allergy prevalence of 15.6%. Our penicillin allergy prevalence of 14.7% is higher than the 8–12% previously reported [1–4]. Penicillin allergy is frequently overdiagnosed and prevalence is highly dependent on the efforts made by providers obtaining an allergy history [17,23,26,29]. In our system, we found striking variability in the beta-lactam allergy prevalence among provider panels. Among providers with a panel size of at least 1000 patients, beta-lactam allergy prevalence ranged by a factor of three (7.8% to 24.8%). The fact that some providers diagnose a beta-lactam allergy more than other providers suggests that many patients are being misclassified as having beta-lactam allergy while others may be misclassified as not having an allergy.

Allergy documentation is frequently incomplete. In our study, only 39.8% of allergens were specifically identified and only 22.7% of allergic reactions had any documentation. Because an allergy history is frequently dependent on patient recall there are times when an allergy history will be unknown or incomplete despite a provider’s best efforts. We found significant variability amongst providers in the rate of identification of specific allergens (24.0% to 58.2%) and documentation of characteristics of the reactions (5.4% to 51.9%). This degree of variability suggests that real opportunities to record these data are often missed.

There is also striking variability in how beta-lactam allergies and the specific components of the allergy history are documented. In our evaluation we determined whether inter-provider variability was greater than that expected by chance by simulating expected frequency distributions for variation in beta-lactam allergy history documentation. In comparing expected with observed frequency distributions we found more variation in beta-lactam allergy, allergen and reaction documentation than would have been expected if provider strategies for identification and documentation were consistent. Interestingly, we found that inter-provider variation in documentation of high risk allergic reaction characteristic was not significant, which may be due to enhanced patient recall of a serious reaction and therefore improved allergy evaluation and documentation.

Documenting an allergy requires conducting the allergy history, critically evaluating the history to determine if the patient is truly allergic and documenting the history in a clear manner so as to guide further clinical decision making. Since none of these components are standardized there is room for variability depending on the provider’s thoroughness in conducting a history, willingness to take a patient’s history at face value, knowledge about evaluating putative beta-lactam allergy, diligence in documentation and appetite for risk. Such variation between providers is consistent with findings in other areas of medicine [31–33].

Documentation has consequences. To evaluate how beta-lactam allergy documentation might affect care, we examined the first choice of antimicrobial used on a patient after an allergy was documented. As expected, combined penicillin and cephalosporin use was higher among patients without a beta-lactam allergy documented (38.5% vs 8.2%, p<0.001). We also found higher use of beta-lactams among patients without a beta-lactam allergy for all age groups and for patients with a diagnosis of cellulitis (S1 and S2 Tables). We additionally found that beta-lactam rechallenge was higher when a specific beta-lactam allergen was identified (10.6% vs 6.5%; p<0.001) and when the characteristics of the reaction were documented (13.4% vs 6.8%; p <0.001). Furthermore, use of alternative agents such as fluoroquinolones and clindamycin was higher in patients with documented beta-lactam allergy, and in beta-lactam allergic patients whose record did not identify a specific agent. These findings suggest that providers are more likely to choose an alternate class when a patient has a beta-lactam allergy and the history is not complete. It is likely that when information is incomplete clinicians feel it is safest to assume a history of high-risk reaction and avoid all beta-lactams. However, beta-lactams are the antibiotic of choice for many infections [18,34], and hesitation to use these agents may result in suboptimal treatment, greater lengths of stay, increased costs of care and increased rates of drug resistant pathogens [15–16,19].

Our study suggests that documentation of beta-lactam allergy—a process with real impact on a patient’s care—requires greater attention and standardization. In our EHR, documentation of allergic reactions involves filling out a free text space for the allergen and allergic reaction. This framework allows for significant variability in how an allergy is represented and no clinical decision support around the conduct of a beta-lactam allergy evaluation. A focused allergy history should include the specific allergen, reaction, temporal relationship of reaction to antibiotic use, age at time of reaction and whether the patient was ever rechallenged with that antibiotic class. In addition, providers should understand how to critically evaluate a patient’s report of an allergy to determine if an allergic reaction indicates a true allergy, intolerance or an unrelated symptom. EHR documentation templates could be redesigned to better encourage complete documentation and assist in the process of allergy evaluation. For example, a template that requires documentation of standardized common allergic reactions upon entry of a new allergy, provides a probability of eliciting an IgE mediated reaction when prescribing other beta-lactams, or provides decision support to determine whether a penicillin skin test or an oral challenge is warranted are all areas that could provide meaningful impact. Standardized education for providers in this area could also improve documentation. Further studies are needed to evaluate the impact of educational programs and EHR-based approaches on beta-lactam allergy documentation and inter-physician variability.

A limitation of this study is that we were only able to detect prescriptions provided by or subsequently recorded by providers within the NorthShore system. In an effort to minimize ‘missed’ antibiotic prescriptions, we limited our study to patients cared for by a PCP employed within this system. In this study, we only considered the first antibiotic prescribed after an allergy was documented. We felt the first subsequent prescription was an adequate indicator of post-allergy prescribing behavior, though it is possible that caution about beta-lactam rechallenge diminishes as time passes after the initial allergic episode. We were not able to discern whether incomplete documentation was a result of the patient not knowing the reaction or the healthcare provider not completing the documentation. However, the marked inter-physician variability suggests that true opportunities for detailed documentation are being missed. In evaluating subsequent antibiotic use, we did not match comparator groups. Instead, we looked at the entire population and limited the underlying difference between patients who do and do not have a beta lactam allergy by limiting our study only to patients who received a subsequent antibiotic. We did also evaluate whether antibiotic prescriptions differed among patients with and without beta-lactam allergy for different age groups (S1 Table) and found that the presence or absence of beta lactam allergy resulted in the prescription of distinct classes of antibiotics regardless of age. Further, we were able to give ourselves greater confidence in evaluating the entire population because the difference between our groups with beta-lactam allergies only had to do with allergy documentation and not any underlying difference in the groups. While we are attributing patients to their PCP, only 17% of allergy documentation was performed by PCPs. While not all of the variability observed here can be attributed to them, this does not diminish the key conclusion that standardization of education and documentation methods is much needed to ensure consistency.

Finally, we did not control for underlying patient-level factors that may have predisposed patients to being classified as having beta-lactam allergy and to having more or less detailed documentation. We elected not to attempt to control for these because key drivers of allergy documentation (e.g. lifetime history of beta-lactam exposure, patient health literacy) were not available. However, to minimize the influence of patient-level factors on inter-physician variability we restricted our analysis of variability to providers with a panel size of at least 1000 patients or 100 patients with a beta-lactam allergy, and our study was carried out among PCPs in a single health system in one geographical region. It is unlikely that the marked inter-physician variability can be accounted for solely by differences in panel composition.

## Conclusion

In this study, we found striking inter-physician variability in documenting beta-lactam allergies. This degree of variability suggests that many patients were likely misclassified as having an allergy, and that opportunities to document reaction details were frequently missed. This inconsistency carries important consequences for patients, inclining providers away from the use of safe, effective, inexpensive first-line agents. In an era of concerns about the cost of care, antimicrobial resistance and a dwindling pipeline of new agents, better allergy assessment and documentation should be a focus of medical training and electronic health record design.

## Supporting Information

S1 Table Among patients who received an antibiotic, first antibiotic prescribed after antibiotic allergy documentation by age group^1,2^. *p < 0.001. ^1^ Rows do not add up to 100 because other antibiotic classes not displayed. ^2^ Only patients who received an antibiotic after allergy documentation were included. For patients who did not have a beta-lactam allergy documented, the first antibiotic prescribed is the first antibiotic received during the study period.
(DOCX)

S2 Table Among unique patients with characteristics of beta-lactam allergy documentation, the first antibiotic received at the first inpatient or outpatient clinical encounter with a diagnosis of cellulitis (by ICD9 code)^1,2^. *p < 0.001; ǂ p < 0.01.^1^ Rows do not add up to 100 because other antibiotic classes not displayed. ^2^ For patients with a beta-lactam allergy, first antibiotic received was after allergy documentation.
(DOCX)
