# Supplementary material for: Documenting Penicillin Allergy: The Impact of Inconsistency
Source: PLoS One. 2016 Mar 16;11(3):e0150514. doi: 10.1371/journal.pone.0150514 (PMC4794183; doi:10.1371/journal.pone.0150514)
Supplement: S1 Table — *p < 0.001. 1 Rows do not add up to 100 because other antibiotic classes not displayed. 2 Only patients who received an antibiotic after allergy documentation were included. For patients who did not have a beta-lactam allergy documented, the first antibiotic prescribed is the first antibiotic received during the study period. (DOCX) [file pone.0150514.s001.docx]

**Supplemental Table 1: Among patients who received an antibiotic, first antibiotic prescribed after antibiotic allergy documentation by age group^1, 2^**

| Documentation (N) | Penicillins (%) | Cephalosporins (%) | Fluoroquinolones (%) | Clindamycin (%) | Vancomycin (%) | | Macrolides (%) |
| --- | --- | --- | --- | --- | --- | --- | --- |
| **Age 18-40 (46,660)** | | | | | | | |
| Beta-lactam allergy (4,994) | 5.2* | 4.2* | 16.3* | 9.4* | 0.9* | 51.8* | |
| No beta-lactam allergy (41,666) | 27.6* | 13.3* | 10.8* | 2.5* | 0.1* | 35.5* | |
| **Age 41-60 (62,027)** | | | | | | | |
| Beta-lactam allergy (7,922) | 3.1* | 4.1* | 23.9* | 10.0* | 1.2* | 48.6* | |
| No beta-lactam allergy (54,105) | 22.3* | 15.9* | 15.5* | 2.6* | 0.2* | 35.8* | |
| **Age 61-80 (39,976)** | | | | | | | |
| Beta-lactam allergy (6,018) | 3.2* | 4.8* | 28.9* | 12.4* | 3.2* | 37.0* | |
| No beta-lactam allergy (33,958) | 17.0* | 20.2* | 20.9* | 3.4* | 0.6* | 29.5* | |
| **Age >80 (12,995)** | | | | | | | |
| Beta-lactam allergy (2,262) | 2.9* | 6.1* | 36.7* | 12.9* | 4.4* | 25.5* | |
| No beta-lactam allergy (10,733) | 13.6* | 21.9* | 28.0* | 4.3* | 1.1* | 21.2* | |

*p < 0.001; ǂ p < 0.01; Ϯ p < 0.05

^1^ Rows do not add up to 100 because other antibiotic classes not displayed

^2^ Only patients who received an antibiotic after allergy documentation were included. For patients who did not have a beta-lactam allergy documented, the first antibiotic prescribed is the first antibiotic received during the study period
