# Supplementary material for: Documenting Penicillin Allergy: The Impact of Inconsistency
Source: PLoS One. 2016 Mar 16;11(3):e0150514. doi: 10.1371/journal.pone.0150514 (PMC4794183; doi:10.1371/journal.pone.0150514)
Supplement: S2 Table — *p < 0.001; ǂ p < 0.01.1 Rows do not add up to 100 because other antibiotic classes not displayed. 2 For patients with a beta-lactam allergy, first antibiotic received was after allergy documentation. (DOCX) [file pone.0150514.s002.docx]

**Supplemental Table 2: Among unique patients with characteristics of beta-lactam allergy documentation, the first antibiotic received at the first inpatient or outpatient clinical encounter with a diagnosis of cellulitis (by ICD9 code)^1, 2^**

| Documentation (N) | Penicillins (%) | Cephalosporins (%) | Fluoroquinolones (%) | Clindamycin (%) | Vancomycin (%) | Macrolides (%) |
| --- | --- | --- | --- | --- | --- | --- |
| **ALL PATIENTS (1,329)** | | | | | | |
| Beta-lactam allergy (265) | 6.8* | 8.7* | 13.6* | 32.1* | 14.3* | 4.5* |
| No beta-lactam allergy (1,064) | 33.1* | 22.5* | 2.6* | 20.9* | 7.0* | 0.7* |
| **WAS A SPECIFIC BETA- LACTAM ALLERGEN IDENTIFIED? (265)** | | | | | | |
| Specific beta-lactam (111) | 11.7ǂ | 9.0 | 10.8 | 30.6 | 16.2 | 2.7 |
| No specific beta-lactam (154) | 3.2ǂ | 8.4 | 15.6 | 33.1 | 13.0 | 5.8 |
| **WERE CHARACTERISTICS OF BETA-LACTAM REACTION DOCUMENTED? (265)** | | | | | | |
| Documented (79) | 8.9 | 8.9 | 12.7 | 25.3 | 25.3* | 1.3 |
| Not documented (186) | 5.9 | 8.6 | 14.0 | 34.9 | 9.7* | 5.9 |
| **WAS BETA-LACTAM REACTION HIGH RISK? (79)** | | | | | | |
| High risk reaction (14) | 14.3 | 0 | 7.1 | 35.7 | 28.6 | 0 |
| Not a high risk reaction (65) | 7.7 | 10.8 | 13.8 | 23.1 | 24.6 | 1.5 |

*p < 0.001; ǂ p < 0.01; Ϯ p < 0.05

^1^ Rows do not add up to 100 because other antibiotic classes not displayed

^2^ For patients with a beta-lactam allergy, first antibiotic received was after allergy documentation
